# Endangered Uyghur Medicinal Plant *Ferula* Identification through the Second Internal Transcribed Spacer

**DOI:** 10.1155/2015/479879

**Published:** 2015-06-15

**Authors:** Congzhao Fan, Xiaojin Li, Jun Zhu, Jingyuan Song, Hui Yao

**Affiliations:** ^1^Xinjiang Institute of Chinese Materia Medica and Ethnical Materia, 9 Xinming Road, Urumqi, Xinjiang 830002, China; ^2^The National Engineering Laboratory for Breeding of Endangered Medicinal Materials, Institute of Medicinal Plant Development, Chinese Academy of Medical Sciences and Peking Union Medical College, Beijing 100193, China; ^3^Chongqing Institute of Medicinal Plant Cultivation, Chongqing 408335, China

## Abstract

The medicinal plant *Ferula* has been widely used in Asian medicine, especially in Uyghur medicine in Xinjiang, China. Given that various substitutes and closely related species have similar morphological characteristics, *Ferula* is difficult to distinguish based on morphology alone, thereby causing confusion and threatening the safe use of *Ferula*. In this study, internal transcribed spacer 2 (ITS2) sequences were analyzed and assessed for the accurate identification of two salable *Ferula* species (*Ferula sinkiangensis* and *Ferula fukangensis*) and eight substitutes or closely related species. Results showed that the sequence length of ITS2 ranged from 451 bp to 45 bp, whereas guanine and cytosine contents (GC) were from 53.6% to 56.2%. A total of 77 variation sites were detected, including 63 base mutations and 14 insertion/deletion mutations. The ITS2 sequence correctly identified 100% of the samples at the species level using the basic local alignment search tool 1 and nearest-distance method. Furthermore, neighbor-joining tree successfully identified the genuine plants *F. sinkiangensis* and *F. fukangensis* from their succedaneum and closely related species. These results indicated that ITS2 sequence could be used as a valuable barcode to distinguish Uyghur medicine *Ferula* from counterfeits and closely related species. This study may broaden DNA barcoding application in the Uyghur medicinal plant field.

## 1. Introduction

The endangered Uyghur medicinal herb* Ferula* has a long history of application in China. It was first utilized in the* Tang Materia Medica* during the Tang Dynasty (AD 659) because of its effectiveness in removing food residues, as well as its dispersed stuffiness and chlordime form [1]. To date,* Ferula* is traditionally employed for treating different diseases such as asthma, epilepsy, stomach ache, flatulence, intestinal parasites, weak digestion, and influenza in many countries [2, 3]. Pharmacological and biological studies have shown that* Ferula* has antioxidant [4], antiviral [5], antifungal [6], cancer chemopreventive [7], antidiabetic [8], antispasmodic [6], hypotensive [9], and molluscicidal effects [10].* Ferula* is now included in the Uyghur Medicine Criteria [11] and calendar version of the Chinese Pharmacopoeia [12]. The original plants for this medicine are* Ferula sinkiangensis *and* Ferula fukangensis. *In addition to a close relationship with traditional Chinese medicine, Uyghur medicine has absorbed the essences of Indian, Arabian, Iran, and ancient Greek medicine, thereby forming a complete system of medicine with distinctive ethnic characteristics. However, its medicinal parts and functions are not precisely the same. For instance, traditional Chinese medicine utilizes* Ferula* resin, whereas Uyghur medicineutilizes* Ferula* resin, roots, and seeds [13] as clinical parts.

In China, the* Ferula *genus is mostly distributed in Xinjiang, but has been destroyed in this area as a result of excessive collection. In addition to seed plant breeding, change in reclamation, irrigation, road building, and deterioration of the original habitat contribute to the annual shrinking of* Ferula* resources. Currently,* F. fukangensis* is on the verge of extinction, and* F. sinkiangensis* is listed as a class 3 endangered plant and class 2 protected wild medicinal species. Given its high cost and scarce resources, various substitutes and adulterants have emerged.* F. sinkiangensis *and* F. fukangensis* have been misused and substituted by other species such as* F. ferulaeoides* Korov [14]. Furthermore, numerous species from the* Ferula* genus have been utilized as traditional Chinese medicines and ethical materials. For example,* F. soongarica* has been utilized by Kazakhs in treating headaches, colds, and stomach aches;* F. caspica* in treating nervous breakdown; and* F. lehmannii *in treating parasitic malnutrition and cold pain in the heart and abdomen [15]. When mixed with substitutes, adulterants, and closely related species, the original medicinal plant could not be traced to the medicinal herb market. A famous line that is widely circulated in the herbal medicine field is “there is no false* Scutellaria*, no true* Ferula.*” Thus, investigating the authenticity of the Uyghur medicinal herb* Ferula *is urgently required.

The* Ferula* genus is usually recognized as monophyletic; the delimitation of species requires examination of complete specimens with roots, stem bases, basal leaves, inflorescence, flowers, and ripe fruits based on the observations of living plants [16]. Given that members are similar in habit and morphology, flowers, inflorescences, and fruit anatomy are often hardly discernible taxa [17, 18]. Thus, the infrageneric classification method of the Uyghur medicinal herb* Ferula* urgently requires a solution. To address this problem, microscopic identification and powder identification techniques have been utilized for* F. sinkiangensis* and its closely related species [19–21]. However, these techniques could not solve the problem of authenticating the Uyghur medicinal herbs* F. sinkiangensis *and* F. fukangensis *from other traditional medicinal herbs and closely related* Ferula *species. By comparison, DNA barcoding employs a short DNA sequence from a standard locus as a species identification tool [22], which has been widely applied in forage identification [23] and animal genetic relationship identification [24]. The internal transcribed spacer (ITS) region of this barcode, which comprises ITS1, 5.8S, and ITS2, has been widely utilized as a core DNA barcode to identify different herbal medicinal materials [25]. Researchers have proposed ITS2 as a standard DNA barcode for medicinal plant authentication [26, 27]. However, molecular identification of* F. sinkiangensis* and* F. fukangensis* from their closely related species utilizing the ITS2 sequence has not been reported. In the present study, DNA barcoding technology was applied for the first time to distinguish* F. sinkiangensis* and* F. fukangensis* from their succedaneum and other closely related species. The application of DNA barcoding in trade and market management could ensure the safe use of the pharmacopoeia herb* Ferula*.

## 2. Samples and Methods

### 2.1. Materials and Methods for Sampling of Plant Materials

Seventy-three leaves and root samples, which represented 10* Ferula* species, were collected from different locations. Nine* Ferula* species were acquired from Xinjiang and one species of* Ferula litwinowiana* was obtained from Kazakhstan. The 10 species were given specimen collection numbers (Table S1) (see Supplementary Material available online at http://dx.doi.org/10.1155/2015/479879) and authenticated by Researcher Guanmian Shen (Xinjiang Institute of Ecology and Geography, Chinese Academy of Sciences) and Associate Fellow Guoping Wang (Xinjiang Institute of Chinese Traditional Medical and Ethical Materia Medica). All corresponding voucher samples were deposited in the herbarium of the Xinjiang Institute of Chinese Traditional Medical and Ethical Materia Medica. In addition, one published sequence was downloaded from GenBank for analysis (Table S1).

### 2.2. DNA Extraction, Amplification, and Sequencing

Genomic DNA was isolated from 30 mg of leaves or roots according to the protocol of the Plant Genomic DNA Kit (Tiangen Biotech Co., China). Specific regions of the ITS2 sequences were amplified from dried leaves and root extracts; universal primer and PCR conditions for ITS2 were obtained from previous studies [28]. The purified PCR products were sequenced using a 3730XL sequencer (Applied Biosystems, Foster City, CA, USA).

### 2.3. Sequence Alignment and Analysis

The CodonCode Aligner v 4.0.4 was utilized to edit the sequences and assemble the contigs and shear sequence using universal primers (5′-3′: ATTCACACCAAGTATCGCAT and 3′-5′: ATTGTAGTCTGGAGAAGCGTC). The ITS2 core region could be obtained using the HMMer annotation method based on the hidden Markov model (HMM) to remove the 5.8S and 28S sections at both ends of the sequences [29]. The interspecific/intraspecific variation of the samples was calculated according to Kress et al. [22]. A phylogenetic tree was constructed utilizing MEGA 5.0. Neighbor-joining algorithm (NJ tree) was used to evaluate the capability of the ITS2 sequence to authenticate the studied species. Bootstrap tests were conducted utilizing 1,000 resampling to assess the confidence of the phylogenetic relationships using employed MEGA 5.0 [30, 31]. BLAST1 and nearest-distance methods were performed to identify species, as previously described [32, 33].

## 3. Results

### 3.1. Universality and Sequence Characteristics

DNA was successfully extracted from 73 samples. Gel electrophoretic analysis revealed that all sample extractions emitted bright bands. ITS2 sequences were successfully amplified and sequenced; high-quality bidirectional sequences were obtained. The ITS2 sequence, including partial the 5.8S, ITS2 region, and partial 28S, ranged from 451 bp to 455 bp, and GC contents were 53.6% to 56.2%. The partial 5.8S length was 84 bp; the GC contents ranged from 57.1% to 58.3%. The length of the ITS2 region was 226 bp to 230 bp; the GC contents ranged from 53.0% to 56.8%. The partial 28S was 141 bp and the GC contents ranged from 51.8% to 53.9%.

### 3.2. Intraspecific/Interspecific Variations

Sequence length, variable sites, and average interspecific K2P distance of ITS2 regions were analyzed and summarized (Table 1). Most of the 10* Ferula* species in this study were represented by severalsamples because they were collected from different localities.* F. sinkiangensis* was represented by 13 samples (Table S1),* F. fukanensis* by 6 samples,* F. ferulaeoides* by 14 samples,* F. soongarica* by 6 samples,* F. caspica* by 11 samples, and so on (Table 1). The results showed that each* Ferula* species only possesses a haplotype, and their intraspecific distances were all zero.

**Figure 1:**
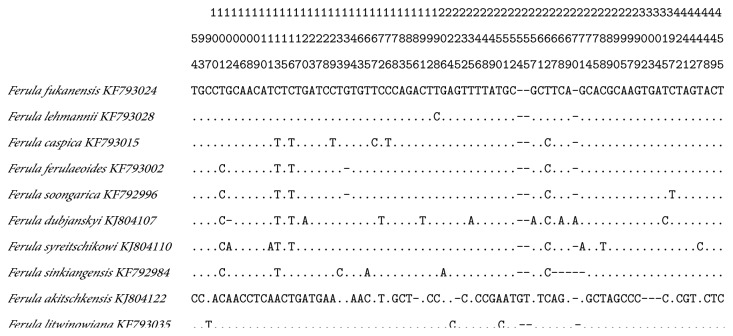
Variable sites in the haplotypes of the 10* Ferula* species.

Among the 74 ITS2 sequences, a total of 77 variation sites were detected in the region, including 63 base mutations and 14 insertion/deletion mutations. The following contents were detected among the 63 base mutation sites: C/T (26), C/G (5), A/C (12), G/A (10), T/A (5), T/G (4), C/G/A (1), and C/T/A (4) substitutions. A total of 76 distinctive variable sites were discovered among the 77 variation sites, which could precisely authenticate different* Ferula* species (Figure 1). For instance, the C–T transition at site 133 and the A–G transition at site 206 could distinguish* F. sinkiangensis* from the othernine* Ferula *species. The C–T transition at the 397 bp site could distinguish* F. soongarica* and the C–T transition at site 176 could individually classify* F. caspica*. The interspecific divergence calculated utilizing the K2P model indicated that the ITS2 sequences of* F. sinkiangensis* possessed an average interspecific distance of 0.030;* F. fukanensis* possessed an average intraspecific distance of 0.027 (Table 1).

### 3.3. Species Identification Capability of ITS2 Barcode and NJ Tree Analysis

BLAST1, nearest distance, and tree-based methods were utilized to determine whether the certified medicinal herb* Ferula* could be identified by ITS2 sequences. The results indicated that ITS2 correctly identified 100% of the samples at the species level through the BLAST1 method. The nearest genetic distance method also resulted in 100% success rate of identification.

**Figure 2:**
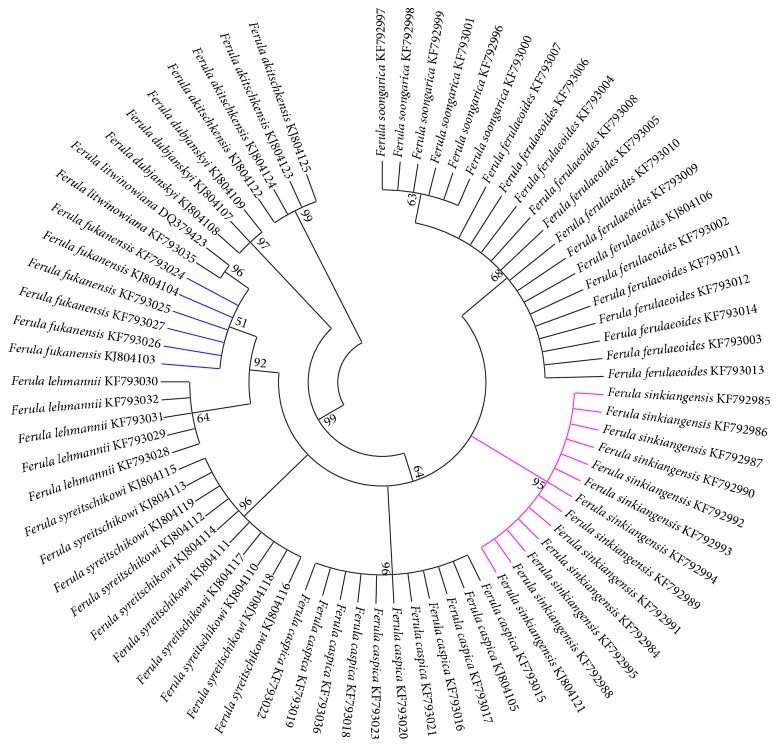
NJ tree of* F. sinkiangensis and F. fukangensis* and their closely related species constructed with the ITS2 sequence. The bootstrap scores (1,000 replicates) are shown (≥50%) for each branch.

Up to 74 ITS2 sequences of* Ferula* species were chosen to build the trees. NJ trees (Figure 2) were constructed based on the ITS2 sequences. The results showed that the Uyghur medicinal herbs* F. fukangensis* and* F. sinkiangensis* presented good monophyly; they could be correctly distinguished from their succedaneum* F. ferulaeoides *and other closely related species. Furthermore, the remaining* Ferula* species could be distinguished from one another. Thus, the ITS2 barcode could correctly identify two Uyghur medicinal herb plants from their adulterants and closely related species.

## 4. Discussion

### 4.1. ITS2 Region Is a Useful DNA Barcode to Differentiate 10* Ferula* Species

The genus* Ferula* is an important ephemeral plant that possesses highly ecological and medicinal values. Our results emphasized the advantages of using the ITS2 region as a DNA barcode, including high interspecific divergence but small intraspecific variation, favorable universality, and short length, which lead to easy amplification and sequencing efficiency. Given that pharmacologists often traded* Ferula* genus plants internationally for their medicinal values, our research provides a convenient tool for validating the quality products of* Ferula*. In a previous study [18], 83 accessions (74 species) of* Ferula *were used to ascertain the phylogenetic position of the genus within the family. Most species had one sample; hence, the intraspecific divergence could not be determined. The ITS1 and ITS2 regions are separately amplified, and then assembled to successfully redress several misplaced* Ferula* genus plants. Phylogenetic analyses of tree-based method successfully corrected the three accessions of misplaced* Ferula* species. In our study, 74 ITS2 sequences of 10* Ferula* species were used to authenticate* F. sinkiangensis and F. ferulaeoides *from their succedaneum and closely related species, most of which were newly reported sequences and each species was represented by two or more samples. Therefore, intraspecific variation was clearly displayed.

Morphological characteristics are an important basis for identifying the original* Ferula *plants. However, morphological identification often relies on abundant experience, which could be easily affected by biocoenosis and geographical environment [17]. As a result, identification of traditional Chinese medicine* Ferula* has been limited. Genomic sequence is not influenced by individual characteristics and developmental stages; therefore, DNA barcode technology has been widely used in recent years [34]. DNA barcoding is an effective supplement to traditional morphological methods, and, in our study, the phylogenetic tree (Figure 2) demonstrated that the ITS2 region has an effective role in authenticating the Uyghur medicinal herb* Ferula* and differentiating 10* Ferula *species from each other.

### 4.2. Identification of Uyghur Medicine Utilizing ITS2 Sequence

According to the records, Uyghur medicine includes 1,000 types of herbal medicines and up to 450 types are frequently utilized. Nevertheless, limited by rare resources and confusing plant origins, only about 200 species of Uyghur medicinal herbs have been formulated based on national and local criteria. Aside from the Uyghur medicinal herb* Ferula*, numerous other Uyghur medicinal herbs, such as* Cichorium intybus* L. and* Cichorium glandulosum* Boiss. et Huet, are facing the same dilemma [14]. These situations are significant obstacles to the standardization and industrialization of Uyghur medicine. Although morphological examination and chemical analysis are routine practices in identifying Uyghur medicinal herbs, these methods are influenced by biological and physical factors [35]. DNA barcoding and forensically informative nucleotide sequencing are less affected by these elements. The ITS2 barcode has been proposed as a universal DNA barcode for medicinal herb and animal identification [26, 27, 36, 37]. Thus, based on this study, DNA barcoding would have a broad prospect in authenticating more Uyghur medicinal herbs, which provides a new method for the Food and Drug Administration to control Uyghur medicine and ensure its safe and efficient use and has a considerable role in developing the Uyghur medicine industry.

### 4.3. Significance of DNA Barcode in Food Safety Control

Although several* Ferula* genus plant roots are used traditional medicine or folk medicine in Xinjiang, few* Ferula* species are edible. Starch extracted from* F. ferulaeoides and F. dubjanskyi* could be made into food [2]. The tender leaves of* F. lehmannii* could be used as wild herbs to make cold dishes and dumplings [38]. DNA barcoding has been used for food safety control [39–41]. The NJ tree demonstrated that the ITS2 barcode could accurately perform food safety control. Sequencing the ITS2 DNA barcode region represents a new technique that guarantees food and drug safety in* Ferula *species. This technology is easy to learn and utilized by food managers [42].

## Supplementary Material

Samples and DNA sequences used in this study

## Figures and Tables

**Table 1:** ITS2 sequence characteristics and K2P distances of *Ferula* used in this research.

Species	Sample number	GC content (%)	Sequence length (bp)	Distinctive variable site	Interspecific K2P distance (mean)
*F. sinkiangensis *	13	55.0	451	7	0.009–0.163 (0.030)
*F. fukanensis *	6	54.7	455	0	0.002–0.161 (0.027)
*F. ferulaeoides *	14	54.6	454	0	0.002–0.150 (0.023)
*F. soongarica *	6	54.4	454	1	0.002–0.154 (0.025)
*F. caspica *	11	54.3	455	3	0.009–0.161 (0.030)
*F. lehmannii *	5	54.9	455	0	0.002–0.157 (0.028)
*F. syreitschikowi *	10	54.3	455	3	0.007–0.145 (0.028)
*F. akitschkensis *	4	56.2	452	53	0.145–0.170 (0.156)
*F. dubjanskyi *	3	53.6	455	5	0.016–0.151 (0.035)
*F. litwinowiana *	2	54.9	455	4	0.007–0.170 (0.033)
