# Supplementary material for: Endangered Uyghur Medicinal Plant Ferula Identification through the Second Internal Transcribed Spacer
Source: Evid Based Complement Alternat Med. 2015 Jun 15;2015:479879. doi: 10.1155/2015/479879 (PMC4451158; doi:10.1155/2015/479879)
Supplement: Supplementary file 1 — Samples and DNA sequences used in this study [file 479879.f1.pdf]

Table S1. The species information of *Ferula* genus used in this study.

| Latin name | Specimen voucher | Sample source | Sampling parts | GenBank accession |
|---|---|---|---|---|
| *Ferula sinkiangensis* K.M.Shen | 7-x-z-7-1 | Yining County, Xinjiang | leaves | KF792984 |
| | 7-x-z-7-2 | Yining County, Xinjiang | leaves | KF792985 |
| | 7-x-z-7-3 | Jeminay County, Xinjiang | leaves | KF792986 |
| | 7-x-z-7-4 | Jeminay County,Xinjiang | leaves | KF792987 |
| | 7-x-z-7-5 | Yining County, Xinjiang | leaves | KF792988 |
| | 7-x-z-8-2 | Yining County, Xinjiang | leaves | KF792995 |
| | 7-x-z-7-6 | Yining County, Xinjiang | roots | KF792989 |
| | 7-x-z-7-7 | Yining County, Xinjiang | leaves | KF792990 |
| | 7-x-z-7-8 | Jeminay County, Xinjiang | leaves | KF792991 |
| | 7-x-z-7-9 | Jeminay County, Xinjiang | roots | KF792992 |
| | 7-x-z-7-10 | Yining County, Xinjiang | leaves | KF792993 |
| | 7-x-z-8-1 | Yining County, Xinjiang | leaves | KF792994 |
| | 13909 | Shawan,County,Xinjiang | roots | KJ804121 |
| *Ferula fukanensis* K.M.Shen | 7-x-z-3-2 | Fukang County, Xinjiang | leaves | KF793025 |
| | 7-x-z-3-5 | Fukang County, Xinjiang | leaves | KF793027 |
| | 7-x-z-3-4 | Fukang County, Xinjiang | leaves | KF793026 |
| | 7-x-z-3-1 | Fukang County, Xinjiang | roots | KF793024 |
| | 13113 | Fukang County, Xinjiang | roots | KJ804103 |
| | 13114 | Fukang County, Xinjiang | roots | KJ804104 |
| *Ferula ferulaeoides* (Steud.) Korov. | 7-x-z-2-4 | Toli County, Xinjiang | roots | KF793002 |
| | 7-x-z-2-5 | Toli County, Xinjiang | leaves | KF793003 |
| | 7-x-z-2-6 | Fuyun County, Xinjiang | leaves | KF793004 |
| | 7-x-z-2-7 | Fuyun County, Xinjiang | leaves | KF793005 |
| | 7-x-z-2-8 | Fuyun County, Xinjiang | leaves | KF793006 |
| | 7-x-z-2-9 | Toli County, Xinjiang | leaves | KF793007 |
| | 7-x-z-2-10 | Shihezi City, Xinjiang | leaves | KF793008 |
| | 7-x-z-2-11 | Shihezi City, Xinjiang | leaves | KF793009 |
| | 7-x-z-2-13 | Toli County, Xinjiang | leaves | KF793010 |
| | 7-x-z-2-15 | Toli County, Xinjiang | leaves | KF793011 |
| | 7-x-z-2-15 | Yumin County, Xinjiang | leaves | KF793012 |
| | 7-x-z-2-17 | Yumin County, Xinjiang | leaves | KF793014 |
| | 7-x-z-2-16 | Qitai County, Xinjiang | leaves | KF793013 |
| | 13912 | Yumin County, Xinjiang | roots | KJ804106 |
| *Ferula soongarica* Pall. ex Spreng. | 7-x-z-9-10 | Jeminay County, Xinjiang | root | KF792997 |
| | 7-x-z-9-9 | Toli County, Xinjiang | root | KF792996 |
| | 7-x-z-9-11 | Wenquan County, Xinjiang | leaves | KF792998 |
| | 7-x-z-9-13 | Wenquan County, Xinjiang | leaves | KF792999 |
| | 7-x-z-9-15 | Jeminay County, Xinjiang | leaves | KF793001 |
| | 7-x-z-9-14 | Toli County, Xinjiang | leaves | KF793000 |

| | | | | |
|---|---|---|---|---|
| *Ferula caspica* M.B. | 7-s-y-9-2 | Toli County, Xinjiang | leaves | KF793015 |
| | 7-s-y-9-3 | Habahe County, Xinjiang | leaves | KF793016 |
| | 7-s-y-9-4 | Toli County, Xinjiang | leaves | KF793017 |
| | 7-s-y-9-12 | Habahe County, Xinjiang | leaves | KF793036 |
| | 7-s-y-9-6 | Toli County, Xinjiang | leaves | KF793018 |
| | 7-s-y-9-8 | Burqin County, Xinjiang | roots | KF793020 |
| | 7-s-y-9-9 | Burqin County, Xinjiang | leaves | KF793021 |
| | 7-s-y-9-10 | Toli County, Xinjiang | leaves | KF793022 |
| | 7-s-y-9-11 | Fuyun County, Xinjiang | leaves | KF793023 |
| | 7-s-y-9-7 | Fuyun County, Xinjiang | leaves | KF793019 |
| | 13880 | Aletai city, Xinjiang | roots | KJ804105 |
| *Ferula lehmannii* Boiss. | 7-s-z-2-1 | Shawan County, Xinjiang | leaves | KF793028 |
| | 7-s-z-2-3 | Shawan County, Xinjiang | leaves | KF793030 |
| | 7-s-z-2-4 | Manas County, Xinjiang | roots | KF793031 |
| | 7-s-z-2-5 | Shihezi City, Xinjiang | leaves | KF793032 |
| | 7-s-z-2-2 | Shihezi City, Xinjiang | leaves | KF793029 |
| *Ferula dubjanskyi* Korov. ex Pavl. | 13889 | Fukang city, Xinjiang | roots | KJ804107 |
| | 13891 | Buerjin County, Xinjiang | leaves | KJ804108 |
| | 13911 | Fukang city, Xinjiang | leaves | KJ804109 |
| *Ferula akitschkensis* B. Fedtsen. | 13855 | Habahe County, Xinjiang | leaves | KJ804122 |
| | 13854 | Yumin County, Xinjiang | leaves | KJ804123 |
| | 13853 | Aletai city, Xinjiang | leaves | KJ804123 |
| | 13852 | Tuoli County, Xinjiang | roots | KJ804125 |
| *Feruia syreitschikowii* K.-Pol. | 13883 | Manasi city, Xinjiang | leaves | KJ804110 |
| | 13857 | Manasi city, Xinjiang | leaves | KJ804111 |
| | 13861 | Urumqi city, Xinjiang | leaves | KJ804112 |
| | 13862 | Urumqi city, Xinjiang | leaves | KJ804113 |
| | 13865 | Urumqi city, Xinjiang | leaves | KJ804114 |
| | 13871 | Shawan County, Xinjiang | leaves | KJ804115 |
| | 13868 | Shawan County, Xinjiang | leaves | KJ804116 |
| | 13876 | Shihezi city, Xinjiang | roots | KJ804117 |
| | 13885 | Shihezi city, Xinjiang | roots | KJ804117 |
| | 13879 | Changji city, Xinjiang | roots | KJ804119 |
| *Ferula litwinowiana* Koso-Pol. | 7-s-y-9-1 | Kazakhstan | roots | KF793035 |
| | | Genbank | unknow | DQ379423 |
